# Antimicrobial Peptides Beyond the Petri Dish: Translational Barriers to Therapeutic Development

**DOI:** 10.1021/acsbiomedchemau.6c00126

**Published:** 2026-09-30

**Authors:** Marc Torrent

**Affiliations:** The Systems Biology of Infection Laboratory, Department of Biochemistry and Molecular Biology, Biosciences Faculty, Universitat Autònoma de Barcelona, Cerdanyola del Vallès, Barcelona 08193, Spain

**Keywords:** antimicrobial peptides, peptide antibiotics, pharmacokinetics, pharmacodynamics, drug delivery, proteolysis, serum protein binding, bioavailability, biofilm, clinical translation

## Abstract

Antimicrobial peptides (AMPs) are promising antibiotics with rapid bactericidal mechanisms, broad-spectrum activity, and efficacy against drug-resistant pathogens. Yet, despite decades of research and development of many active AMPs, translation to the clinic remains limited. Although AMPs kill bacteria efficiently under optimized conditions, the same properties that enable membrane disruption - cationic charge, amphipathicity, and hydrophobicity - also cause their sequestration, degradation, and inactivation in biological fluids and tissues. This review examines how the major administration routes shape AMP pharmacokinetics and identifies the barriers that must be overcome for effective clinical translation. Each route imposes different limitations: oral delivery is limited by proteolytic degradation, bile sequestration, and poor absorption; intravenous administration by plasma protein binding, proteolysis, restricted tissue penetration, and nephrotoxicity; inhaled delivery by sequestration and inactivation in mucus and pulmonary surfactant; and topical delivery by impaired diffusion, sequestration, and proteolysis within wound and biofilm matrices. These limitations should be addressed from the outset of AMP design, with clear strategies on how to control serum binding, protect against protease digestion, and optimize charge and hydrophobic moment, to off–target interactions and toxicity. A pharmacologically informed approach that incorporates these barriers as design constraints is essential to transform AMPs into clinically effective drugs.

## Graphical abstract

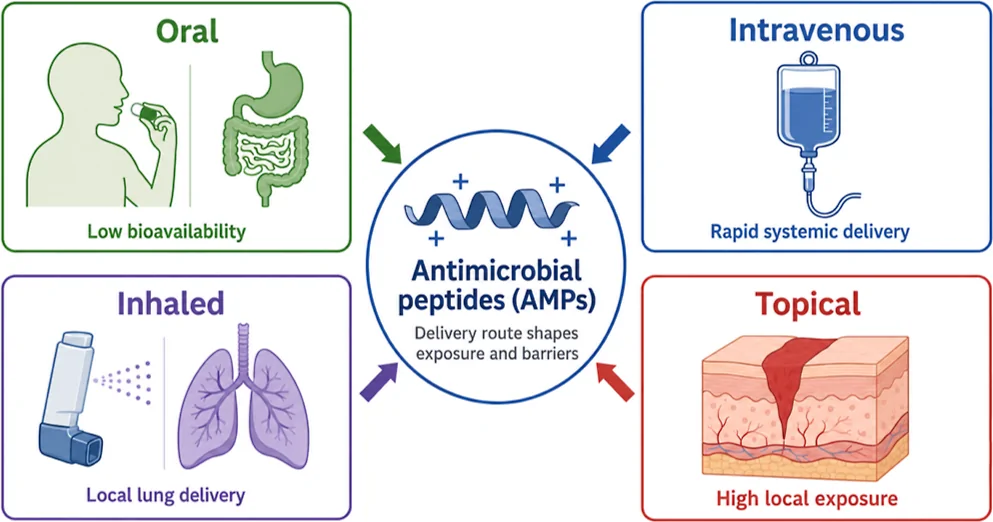

## 1 Introduction

Antimicrobial peptides (AMPs) are promising therapeutic candidates for infections that are difficult to treat.^1–3^ Unlike most classical antibiotics, which act on defined molecular targets, AMPs often kill bacteria by rapidly disrupting membranes, although they can also act through intracellular, cell-wall-targeting, or immunomodulatory mechanisms.^4–6^ Many classical AMPs are small, cationic, and amphipathic, and their antimicrobial activity and selectivity follow defined structure–activity rules, making them engineerable scaffolds for rational sequence optimization.^7–10^ However, clinical translation has remained limited relative to the large number of reported AMP candidates with potent in vitro activity.^3^ Paradoxically, the same properties that enable antimicrobial function can also create important pharmacological barriers. AMPs can bind plasma components (serum proteins, lipoproteins), tissue barriers (extracellular matrices, mucus, pus), biofilm polymers, and host-cell membranes. They can also be degraded by proteases, filtered by the kidneys, metabolized by the liver, or neutralized by immune system proteins, sometimes even before reaching the site of infection. As a result, strong in vitro antimicrobial potency does not necessarily translate into effective activity in vivo.

A central factor in AMP performance is the route of administration.^11^ Compared with small-molecule antibiotics, AMP pharmacokinetics are often more strongly constrained by local biological barriers. AMPs have poor intestinal permeability and are exposed to acidic conditions, bile salts, and digestive proteases. Therefore, oral administration is generally limited to local gastrointestinal infections unless formulation strategies are used to improve stability and absorption.^12^ Intravenous administration bypasses gastrointestinal degradation but also entails other constraints, such as plasma protein binding, lipoprotein sequestration, proteolysis, and exposure to renal and hepatic elimination. Thus, for systemic infections, effective exposure depends not only on the injected dose but also on the fraction of intact, free peptide that can reach bacteria.^13^

Local administration, including topical delivery, can partially overcome these limitations when the site of infection is anatomically localized.^14^ Inhalation can generate high concentrations in the respiratory tract while reducing systemic exposure. This is particularly useful for lung infections such as chronic infections assoiated with cystic fibrosis or bronchiectasis.^15^ Yet, inhaled AMPs must remain active in the airway surface liquid, which cannot be assumed. Similarly, topical administration is well suited for infected wounds, burns and ulcers, and device-associated infections, where AMPs can be confined to the infected surface.^16^ Although topical delivery has been one of the most extensively explored routes for AMP administration, it also faces important challenges, such as impaired diffusion through necrotic tissue or biofilms and local cytotoxicity toward host cells required for tissue repair.

In this review, the term AMP is used broadly to include natural and synthetic AMPs, AMP-derived analogues, and selected peptide antibiotics used as clinically informative comparators. The major administration routes (oral, intravenous, inhalational, and topical administration) are examined in terms of how they shape AMP absorption, distribution, local and systemic exposure, inactivation, clearance, and toxicity. Rather than treating pharmacokinetics as a generic property, the emphasis is placed on how specific AMP features determine route-dependent success or failure. Understanding these relationships is essential for the rational design of AMPs because optimization requires balancing antimicrobial potency with stability, tissue access, controlled binding, reduced host toxicity, and appropriate clearance. The last section integrates these barriers into practical design principles for route-specific AMP optimization.

## 2 Oral Administration

Oral administration of peptides for systemic use is usually discouraged because oral bioavailability is extremely poor, often below 1%.^12^ When peptides are administered orally, they first reach the stomach and, after gastric digestion, are transported to the intestine, where they must be absorbed to enter the bloodstream (Figure 1). If the peptide is not absorbed, it is degraded into smaller fragments, sequestered within luminal matrices, metabolized by the intestinal microbiota, or eliminated in feces. Several factors prevent AMPs from reaching systemic circulation after oral administration, the most important being conformational stability, proteolytic degradation, and impaired intestinal absorption.^12^

**Figure 1:**
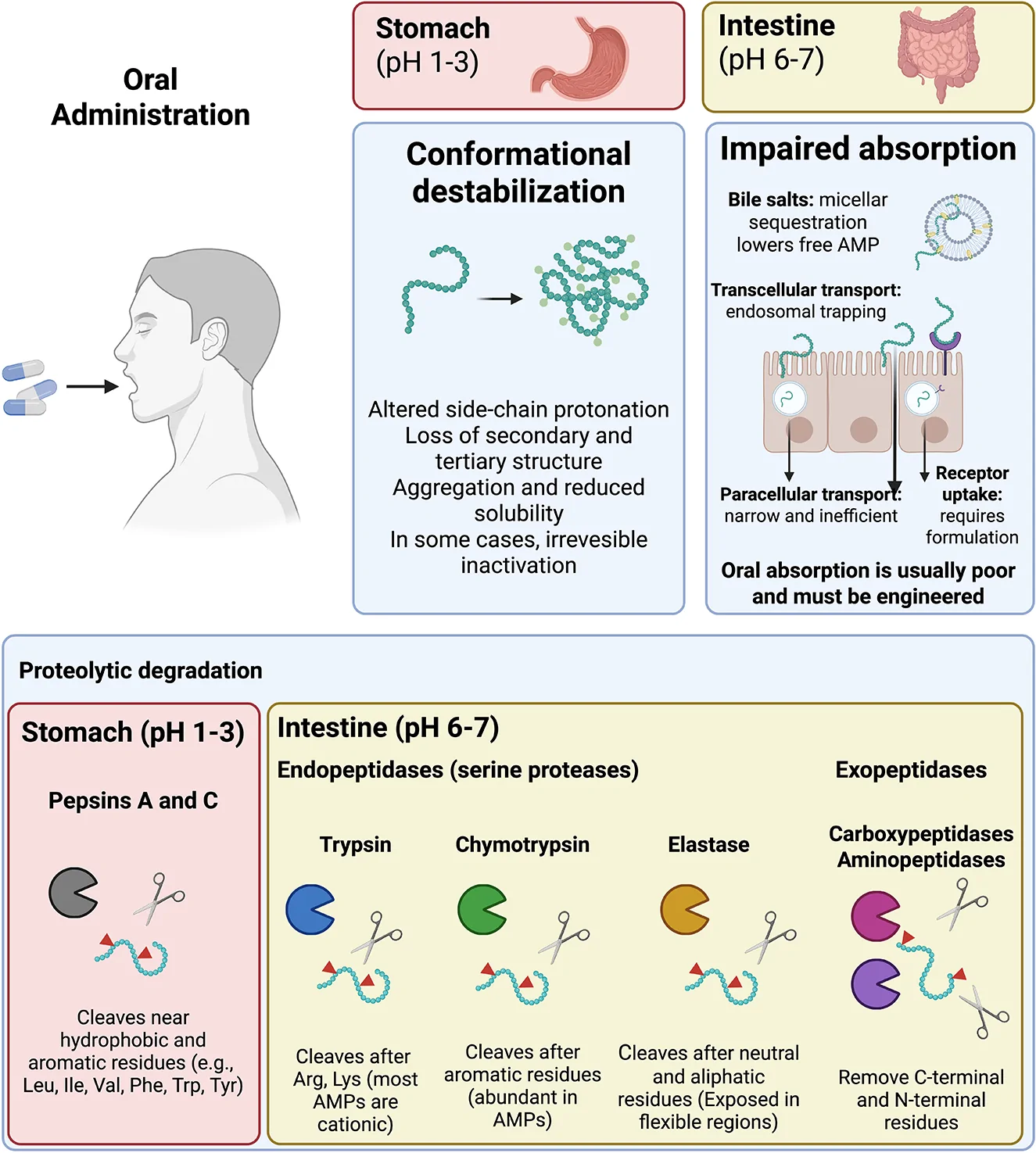
After oral administration, AMPs encounter several barriers in the gastrointestinal tract. In the stomach, acidic pH can alter side-chain protonation, disrupt secondary or tertiary structure, promote aggregation, and cause irreversible inactivation. In the intestine, absorption is limited by bile salt-mediated sequestration, poor paracellular permeability, inefficient transcellular trafficking, and the general need for engineered receptor-mediated uptake. In parallel, gastrointestinal proteases drive extensive degradation. Together, these processes explain why most AMPs have very low oral bioavailability. Created in https://BioRender.com.

### 2.1 Conformational Stability

When peptides reach the stomach, acidic pH can alter side-chain protonation, solubility, secondary or tertiary structure, and aggregation state (Figure 1).^12^ In many cases, AMPs do not adopt a defined secondary structure until they bind to bacterial membranes. However, for AMPs with intrinsic structure, induced conformational changes may become irreversible if they lead to aggregation, reduced solubility, or the formation of inactive conformers. Several cases of inactivation at very low pH have been reported, including some plantaricins, which were irreversibly inactivated after incubation at acidic pH.^214^ In these cases, low pH may lead to irreversible conformational destabilization, aggregation, or loss of solubility.^17^

Nisin, a lantibiotic bacteriocin, provides an instructive counterexample. Although it is not intended for oral administration, it is used as a food preservative and can therefore be orally ingested. Evidence from simulated gastric environments suggests that nisin is extremely stable at pH 3 and loses less than 5% of its activity under these conditions.^18^ This suggests that some peptide structures can be relatively resistant to gastric conditions, although such cases are uncommon. However, during simulated gastrointestinal digestion, nisin was completely degraded by pancreatin.^18^

### 2.2 Proteolytic Degradation

Degradation by proteases in the gastrointestinal tract is one of the principal barriers of oral AMP administration (Figure 1). Several studies suggest that acidic pH and mechanical forces during gastric transit contribute little to peptide degradation on their own, and protease activity is the dominant factor.^17,19^ In the stomach, pepsin A and pepsin C are the main proteases. Pepsin has broad cleavage specificity but preferentially cleaves near aromatic and bulky hydrophobic residues. This is particularly relevant for AMPs because a high fraction of their residues are hydrophobic and therefore susceptible to pepsin-mediated cleavage. Thus, residues that often contribute to membrane insertion, such as Leu, Ile, Val, Phe, Trp, and Tyr, are often targeted by pepsin.^20^ In addition, the low pH of the stomach can promote partial or complete denaturation of the peptide structure, exposing normally hidden cleavage sites and favoring peptide degradation.

When peptides reach the duodenum and enter the intestine, the pH increases to approximately 6–7, and pepsin is rapidly inactivated. The peptide then encounters a sequential proteolytic cascade. First, endopeptidases belonging to the serine protease family (trypsin, chymotrypsin, and pancreatic elastase) cleave the peptide at distinct sites.^21^ Trypsin cleaves on the C-terminal side of cationic residues, Arg and Lys, an important liability for AMPs, which often carry a moderate-to-high positive charge. Chymotrypsin, in turn, mainly cleaves after aromatic residues, which are also frequent in AMPs, whereas pancreatic elastase cleaves after neutral and aliphatic residues, such as Ala, Val, and Gly, which often occur in flexible regions. Together, these proteases cover much of the sequence space of therapeutic peptides. AMPs are also exposed to carboxypeptidase A, which removes C-terminal hydrophobic or aromatic residues, and carboxypeptidase B, which removesbasic residues.^21^

Brush-border peptidases can also contribute to peptide digestion. Important peptidases include aminopeptidases, dipeptidyl peptidase IV, endopeptidase 24.11, and other carboxypeptidases such as carboxypeptidase P.^22^ Ex vivo intestinal models, including Ussing chamber studies, show that brush-border peptidases can rapidly degrade peptides and that transepithelial recovery of intact peptides is often very low. In these models, less than 5% of any given peptide reached the basolateral side after 120 min^23^ Although no consensus half-life exists for AMPs in the digestive tract, the small-intestinal transit occurs over approximately 1–6 h in humans. Therefore, if an unprotected AMP has a half-life of minutes, it will be extensively degraded before absorption can occur.

### 2.3 Impaired Absorption

Even if AMPs remain intact during gastric transit, they must still be absorbed to enter the bloodstream (Figure 1). Within the intestinal lumen, several biochemical factors can interfere with this process. First, bile may pose a significant barrier to AMP absorption. It contains bile acids, phospholipids, cholesterol, bilirubin, electrolytes, and other components that are secreted into the duodenum, where bile salts can reach millimolar concentrations. These bile salts are amphipathic and negatively charged at intestinal pH.^24^ Because most AMPs are also amphipathic and positively charged, electrostatic and hydrophobic interactions can occur between AMPs and bile salts, thereby causing micellar sequestration and reducing the free peptide concentration, potentially affecting activity and bioavailability.^25^ They may also disrupt peptide secondary structure, promoting inactive conformations or aggregation. In simulated gastrointestinal digestion, nisin showed moderate binding to bile salts, producing large micelles that severely reduced its bioavailability.^18^ The effect of bile salts is difficult to predict. They may shield peptides from protease degradation, increasing therapeutic activity, but they may also sequester the peptide and lower the effective concentration below therapeutically active levels.^25^ In some cases, bile salts can also increase permeability by locally perturbing epithelial membranes and affecting tight junctions, facilitating peptide entry into the bloodstream.^26^ Unfortunately, only a few cases of AMP-bile interactions have been studied, and bile effects should be treated as peptide- and formulation-dependent rather than as a uniform barrier.

Free, active AMPs can be absorbed and enter the bloodstream through paracellular absorption or transcellular uptake, including receptor-mediated transcytosis. Paracellular absorption occurs via movement between adjacent epithelial cells, across tight junctions. This route is narrow, size-limited, and charge-sensitive. AMPs are generally too large, too polar, and too charged for efficient, passive paracellular absorption without permeation enhancers.^27^ Another route is transcellular uptake, in which the peptide enters the epithelial barrier from the apical side and exits from the basolateral side. Although transcellular uptake is mechanistically possible, it is generally inefficient as a route to systemic exposure. Transcellular uptake of peptide fragments can occur through PepT1/SLC15A1, the major proton-coupled transporter for dietary dipeptides, tripeptides, and selected peptidomimetic drugs at the intestinal brush border. However, PepT1 is not a receptor and does not provide an efficient route for intact full-length AMPs, whose size, charge, amphipathicity, and conformational properties place them outside the normal substrate range of this transporter.^28^ This route can also be accessed by cell-penetrating peptides, which can cross membranes and reach the cytoplasm.^29^ However, AMPs may be internalized but remain trapped in endosomes or degraded in lysosomes, precluding their transit into the bloodstream.^30^ Receptor-mediated transcytosis exploits endogenous transport systems, but native AMPs do not typically bind intestinal receptors in a way that promotes this process. Other receptor-, transporter- or cell-mediated routes, including vitamin B12/cubam, folate receptor, neonatal Fc receptor (FcRn), apical sodium-dependent bile acid transporter (ASBT), transferrin receptor, and M-cell uptake, have been explored for oral peptide delivery, but these generally require conjugation or encapsulation in nanoparticles or liposomes.^31^ Nevertheless, some substrates can use this mechanism, including β-lactam antibiotics and prodrugs such as valacyclovir.^32^

Thus, for AMPs to be absorbed and enter the bloodstream, engineered systems are generally required, including conjugation, prodrug design, permeation enhancer or encapsulation. Some experimental vancomycin formulations using Labrasol have shown increased intestinal absorption in rats.^33^ This increase is probably due to mixed mechanisms, including partial tight-junction modulation and membrane fluidization. Folic acid-coated liposomes increased oral vancomycin absorption from 2% to 22% via folate receptor-mediated transcytosis.^34^ Microparticle formulations of polymyxin B have also been developed to increase oral polymyxin absorption by targeting *M*-cell uptake in Peyer’s patches, yielding more sustained serum concentrations than free polymyxin.^35^ Therefore, oral absorption of AMPs is usually not an intrinsic property of the free peptide, but a property that must be engineered through formulation.

Despite these barriers to intestinal absorption, AMPs can also be used to treat intestinal infections or formulated as mouthwashes for oral infections. In these cases, systemic absorption is not required, and many of the limitations described above do not apply. For example, vancomycin can be administered orally as a local gut antibiotic, mainly to treat infections, but it does not reach the bloodstream in healthy adults and urinary recovery is negligible.^36^ Colistin sulfate can also be administered orally to treat gastrointestinal infections and shows very poor absorption and almost undetectable levels in plasma. Teicoplanin, although not approved by the FDA for this indication, is also used in several regions as an oral solution for infections and remains undetected in serum or urine at normal doses.^37^ Other classical and modified AMPs, such as ramoplanin, surotomycin, LFF571, and NVB302, are or have been evaluated in clinical trials, and available data suggest that they are not absorbed, although information for some remains limited.^38,39^ However, absorption may occur if the gut is inflamed and the epithelial barrier is compromised. In the case of mouthwashes for local oral or oropharyngeal infections, such as the approved drug tyrothricin, or clinical-stage antibiotics such as histatin, P113, PAC113, and C16G2, the activity is local only, and no absorption through buccal tissues is expected.^40–42^ Tyrothricin, for example, is used locally in the oral and oropharyngeal cavity in some formulations.^40^

## 3 Intravenous Administration

When AMPs enter the bloodstream through intravenous administration, they must overcome five major barriers for effective treatment of infection: distribution, proteolysis, tissue penetration, excretion, and immune-mediated elimination. To exert antimicrobial activity, AMPs must reach the site of infection at sufficient concentrations and remain active long enough to eliminate the pathogen. Consequently, proteolysis, renal or hepatic elimination, and immune clearance must not reduce peptide concentrations below therapeutic levels prematurely.

### 3.1 Plasma Distribution and Unbound Active Concentration

AMPs can bind circulating plasma proteins, causing both beneficial and detrimental effects (Figure 2). On the one hand, protein binding can protect peptides from degradation and excretion, thereby prolonging residence time in the body. On the other hand, extensive protein binding reduces the free, effective concentration and may render the peptide ineffective if free levels fall below the minimum active threshold.^43^ The most important plasma proteins involved in AMP binding are albumin and α1-acid glycoprotein and, to a lesser extent, fibrinogen, α1-antitrypsin, and other serum proteins. Albumin is by far the most relevant AMP-binding protein. With a serum concentration of approximately 35–50 g/L, it is the most abundant plasma protein.^44^ Quantitative ITC studies showed that AMPs can bind albumin with dissociation constants in the low micromolar range, and albumin supplementation increased the MIC of most peptides, consistent with sequestration of the active free fraction.^45^ Albumin contains several hydrophobic binding pockets, including Sudlow sites I and II and fatty acid binding sites FA1-FA7, negatively charged surface patches (pI ∼ 4.7), and a free cysteine residue (Cys34) capable of forming disulfide bonds.^46^ Initially, AMPs can bind electrostatically to negatively charged regions and then hydrophobic regions of the peptide may interact with Sudlow or fatty acid binding sites. Additional polar and van der Waals interactions can further stabilize the AMP–albumin complex.^45^ A detailed study of lactoferricin-derived short AMPs showed, using competitive ITC and NMR WaterLOGSY, that these peptides bind human serum albumin at Sudlow site II and the FA3-FA4 fatty-acid/drug-binding cleft. Docking simulations further supported this model by placing the hydrophobic peptide groups within the Sudlow site II/FA3-FA4 pocket, overlapping with known site-II ligands.^47^ Similarly, dalbavancin, a cyclic lipoglycopeptide antibiotic, binds to the Sudlow site I, and suggests a local conformational adaptation involving Phe70 and a neighboring helix.^48^

**Figure 2:**
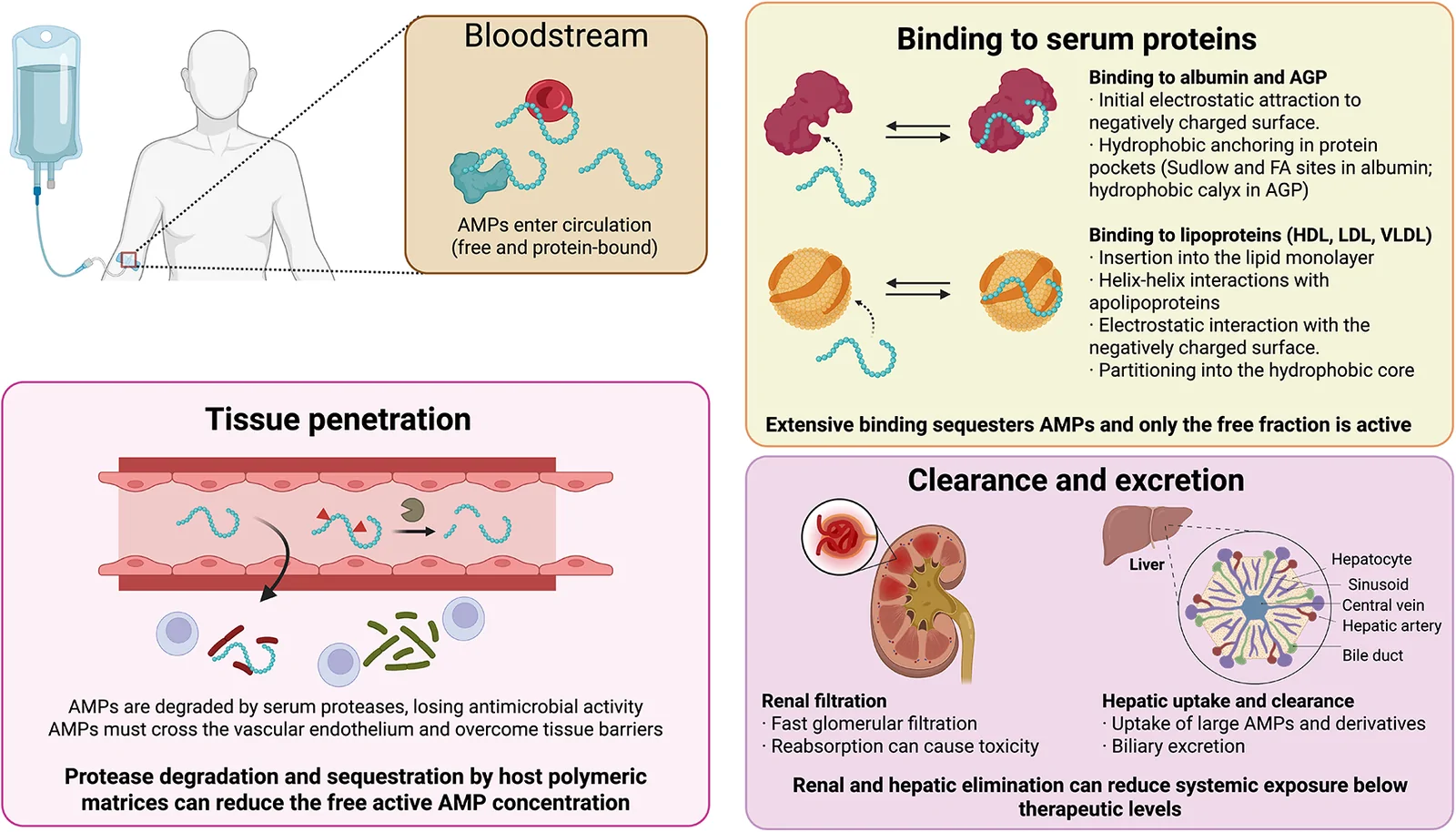
After intravenous administration, AMPs enter the bloodstream as free molecules where they can bind to plasma components. Binding to serum proteins, particularly albumin and α1-acid glycoprotein, and to lipoproteins such as HDL, LDL and VLDL can prolong circulation but also sequester the peptide, reducing the free, active fraction. To reach infected tissues, AMPs must cross the vascular endothelium and diffuse through the extracellular matrix, where proteases and anionic host polymers can also degrade or retain the peptide. Systemic exposure is further limited by renal filtration, hepatic uptake, and biliary elimination. Together, all these mechanisms control whether a sufficient concentration of active AMP can reach the infection site. Created in https://BioRender.com.

AMPs can also bind extensively to α1-acid glycoprotein (AGP), a major acute-phase plasma protein that plays a significant but often underappreciated role in AMP pharmacokinetics.^49,50^ AGP consists of a 22 kDa protein backbone and a heavily glycosylated carbohydrate moiety. Structurally, it contains an eight-stranded antiparallel β-barrel forming a central hydrophobic pocket with five highly sialylated N-linked glycan chains that confer a strong negative charge.^51,52^ AGP concentrations increase substantially during the acute-phase response and may reach over 3 g/L.^53^ Cationic AMPs are attracted electrostatically to the negatively charged glycan chains and, following this initial interaction, the hydrophobic portion of the peptide can insert into the hydrophobic calyx of the β-barrel.^50,54^ The calyx opening is approximately 10 Å wide and 15 Å deep, favoring binding of linear or moderately structured amphipathic peptides rather than large globular proteins.^52^ AGP glycans may further modulate AMP binding by shaping the local electrostatic environment and conformational dynamics near the ligand-binding pocket. This mechanism is supported by desialylation studies showing altered ligand affinity and by molecular-dynamics models indicating that AGP glycans can influence protein dynamics around the ligand-binding site.^55^ Polymyxins provide the clearest example. Binding studies with colistin, polymyxin B, and polymyxin B3 show moderate AGP binding (Kd values typically ∼100–400 μM), whereas derivatives lacking accessible diaminobutyric acid (Dab) positive charges or the N-terminal fatty acyl chain show little or no detectable binding. Molecular modelling places the fatty acyl chain and hydrophobic d-Phe6-l-Leu7 region near the AGP ligand-binding cavity, supporting a mechanism in which electrostatic recognition and hydrophobic anchoring both contribute to AGP binding.^54^ Consequently, high AGP levels during sepsis or systemic inflammation could substantially reduce free polymyxin concentrations, potentially compromising antimicrobial efficacy. Short synthetic AMPs can also bind human AGP with moderate affinity, and docking studies place these peptides within subpockets I–III of the AGP ligand-binding cavities. However, in these cases, AGP binding had little effect on in vitro antibacterial activity, probably because of a low stoichiometric binding ratio.^50^

Although AMPs can also bind plasma proteins other than albumin and AGP, the available evidence suggests that these interactions follow similar physicochemical principles. Cationic charge, amphipathicity, hydrophobic clustering, and peptide conformational flexibility can all contribute, but their relative importance depends on the carrier protein. For example, human neutrophil α-defensins bind fibrin/fibrinogen-associated surfaces, modulate fibrinolysis, and can induce polymeric fibrinogen/thrombospondin-1 amyloid-like assemblies.^56^ α-Defensins also form complexes with serpins, including α1-antitrypsin, and activated α2-macroglobulin has been identified as a major HNP-1-binding protein in plasma.^57^

Stereochemistry can also define how peptides interact with serum proteins. Introducing d-amino acids or converting a peptide entirely into its d-enantiomer changes the three-dimensional arrangement of its side chains and may therefore alter its affinity for circulating proteins. The consequence can be substantial. Lower serum protein binding would leave a larger fraction of peptide unbound and available to exert antibacterial activity. In the case of the LL-37-derived lipopeptide C10-KR8, for example, the L-form associated with a broad range of serum proteins, including several apolipoproteins, whereas the d-enantiomer interacted with markedly fewer proteins and preserved considerably greater antibacterial activity in serum.^58^ Chirality should thus be viewed not only as a determinant of proteolytic stability, but also as a potential regulator of plasma protein binding and peptide distribution.

In addition to soluble plasma proteins, AMPs can also bind circulating lipoprotein particles such as HDL, LDL, and VLDL (Figure 2). These particles can sequester AMPs and substantially reduce the free peptide fraction. Four major mechanisms contribute to AMP-lipoprotein interactions, depending on both peptide and lipoprotein properties. The first mechanism involves partition of the amphipathic helix into the phospholipid monolayer. This is common for α-helical AMPs such as LL-37, magainins, and cecropins, and resembles their interaction with bacterial membranes, although lipoproteins possess a phospholipid monolayer rather than a bilayer.^59,60^ In this case, the cationic face of the AMP interacts with phospholipid headgroups while the hydrophobic face inserts shallowly into the acyl-chain region. The peptide typically lies parallel to the surface, resulting in reversible surface adsorption.^59,60^ The second mechanism involves helix–helix interactions with apolipoproteins and is particularly relevant for HDL particles because of their high content in apolipoproteins. Apolipoproteins such as ApoA-I contain tandem amphipathic α-helices positioned on the lipoprotein surface, with hydrophobic faces embedded in the phospholipid monolayer and polar faces exposed to plasma. Hydrophobic faces of AMP helices can pack against apolipoprotein helices, while electrostatic complementarity provides additional stabilization. This interaction is stereospecific and depends on helix periodicity and hydrophobic moment alignment.^60,61^ The third mechanism involves electrostatic interactions with phospholipid headgroups and apolipoproteins. Lipoproteins possess an overall negative surface charge at physiological pH mainly because of phosphatidylserine, phosphatidylinositol, and negatively charged apolipoprotein residues. Consequently, cationic AMPs are electrostatically attracted to these surfaces. This interaction, however, is generally weak and non-specific.^59^ The last mechanism involves insertion into the hydrophobic lipid core and is particularly important for highly hydrophobic or lipidated AMPs. In this case, the peptide inserts into the phospholipid monolayer. This process is driven largely by the peptide octanol–water partition coefficient (apparent log*P*). VLDL particles and chylomicrons are especially susceptible because of their large hydrophobic cores and relatively fluid monolayers. Polymyxins and other lipopeptide AMPs are particularly prone to this mechanism owing to their lipid tails.^215^

### 3.2 Plasma Proteolysis and Loss of Intact Peptide

Although protein and lipoprotein binding regulate the concentration of free active peptide, the remaining free AMP fraction will be susceptible to proteolytic degradation (Figure 2). Proteolysis can rapidly reduce AMP concentrations and compromise antimicrobial efficacy. Degradation often begins with terminal trimming by exopeptidases, particularly when peptides lack protected termini. N-terminal trimming is mediated by aminopeptidases such as aminopeptidase N, which sequentially remove residues from the N-terminus.^62^ In addition, peptides containing DPP4 recognition motifs, especially X-Pro or X-Ala sequences near the N-terminus, may undergo cleavage by soluble DPP4, resulting in removal of N-terminal dipeptides.^63^ C-terminal trimming can also occur through enzymes such as carboxypeptidase N, which preferentially removes terminal Lys or Arg residues.^64^ This is particularly relevant for AMPs enriched in basic C-terminal residues. In parallel with terminal trimming, AMPs may undergo endoproteolytic cleavage that rapidly disrupts antimicrobial activity. Major plasma endoproteases include plasmin, thrombin, factor Xa, and plasma kallikrein.^65^ In healthy individuals, plasma contains abundant protease inhibitors, including α1-antitrypsin, α2-macroglobulin, and antithrombin III, which normally limit uncontrolled proteolysis.^66^ However, during infection or severe inflammation, these inhibitors can be rapidly consumed or locally inactivated by invading pathogens and activated immune cells, potentially leading to unchecked proteolytic damage.^67^ There are numerous examples in which protease cleavage reduces the antimicrobial action of AMPs.^7,68–70^ For example, plasmin cleaves LL-37 within minutes, and group A *Streptococcus*can recruit host plasminogen to its surface, where streptokinase-generated plasmin degrades bacterium-bound LL-37, increases resistance to cathelicidin-mediated killing, and promotes skin lesion formation in vivo.^71^ Another linear AMP, Pep05, is cleaved preferentially in its N-terminal region, particularly at Lys/Arg and hydrophobic residues such as Leu and Tyr.^72^ Apidaecin Api88 and oncocin, two proline-rich AMPs, are also rapidly degraded in serum, with Api88 undergoing α-thrombin-mediated cleavage and oncocin being cleaved preferentially at basic residue-rich regions.^69^

### 3.3 Penetration into Infected Tissues

After leaving the bloodstream, AMPs must penetrate the infected tissue and retain sufficient free active concentration within the infection site (Figure 2). This step is often limited by endothelial transport, stromal binding, inflammatory debris, biofilm matrices, intracellular localization, and organ-specific barriers.

To leave the bloodstream, AMPs must cross the vascular endothelium and diffuse through the extracellular matrix, with paracellular extravasation being the major route for small peptides.^73^ In this process, peptides pass between endothelial cells through intercellular junctions, mediated by VE-cadherin adherens junctions, which regulate vascular permeability. During infection and inflammation, cytokines and inflammatory mediators disrupt endothelial junctional organization, increasing vascular permeability and allowing plasma proteins and small solutes to enter tissues.^74^ AMPs with small hydrodynamic radii generally extravasate more efficiently because they diffuse more readily through endothelial gaps.^73^ Consequently, small peptides in the low-kDa range are well suited for paracellular transport. In contrast, modifications such as PEGylation, oligomerization, or nanoparticle encapsulation, as well as strong albumin binding, may reduce extravasation efficiency.^75^ Similarly, excessive cationic charge may also impair tissue penetration because the endothelial glycocalyx is highly enriched in negatively charged molecules such as heparan sulfate, chondroitin sulfate, and sialylated glycoproteins,^76^ which can strongly bind AMPs and slow down movement across the endothelial barrier.

Abscesses, purulent exudates, necrotic tissue, and biofilms create combined diffusion and sequestration barriers. These compartments contain bacteria, immune cells, cellular debris, extracellular DNA, membrane fragments, and proteases and are often enclosed within an inflammatory capsule.^77^ Even if an AMP reaches the abscess, penetration into the core may be slow, while negatively charged debris and membrane fragments can act as sinks that trap AMPs, reducing effective concentrations.^78^ Similarly, biofilms constitute major diffusion barriers. The negatively charged extracellular polymeric matrix can slow AMP penetration and bind cationic peptides, thereby reducing antimicrobial activity.^79,80^

Intracellular infections present an additional challenge because AMPs must penetrate host cells and accumulate intracellularly at bactericidal concentrations.^81^ Many AMPs interact efficiently with bacterial membranes but do not accumulate sufficiently within host cells. Moreover, peptides internalized by endocytosis may become trapped within endosomes or lysosomes, where they are degraded by host proteases before reaching intracellular bacteria.^82^ Although increasing peptide hydrophobicity may enhance cellular uptake, this strategy may also increase toxicity, including membrane damage and hemolysis. Short cationic peptides such as WR12 and D-IK8 can reduce intracellular *Staphylococcus aureus*burden in keratinocytes, but the stronger activity of the d-amino-acid peptide D-IK8 illustrates how protease resistance enhances intracellular activity following uptake.^83^ In a similar vein, disulfide-stabilized defensin-like peptides such as NZ2114 have stable tertiary structures that support intracellular killing of *S. aureus* while reducing non-specific mammalian membrane damage.^84^ Correct subcellular localization is also critical for intracellular bactericidal activity. For example, Temporin-SHa penetrates macrophages and localizes in the cytoplasm, but does not enter the vacuolar compartment, leaving residing within vacuoles partly protected from direct peptide action.^85^

Tissue accessibility varies substantially among organs. In highly perfused organs such as the liver and spleen, peptides distribute efficiently but may also undergo rapid clearance by endothelial cells, macrophages, or hepatocytes.^86^ In the kidney, small peptides may be efficiently filtered through the glomerulus, reducing systemic exposure while increasing local renal accumulation, with consequences for both efficacy and nephrotoxicity. In the lung, AMPs must traverse vascular and interstitial barriers and subsequently encounter mucus, surfactant, extracellular DNA, epithelial barriers, and protease-rich inflammatory secretions. These components can sequester or inactivate cationic AMPs, thereby reducing effective concentrations at the infection site.^87,88^ Consequently, the same AMP may exhibit markedly different pharmacological behavior depending on whether the infection is localized in blood, lung, liver, kidney, skin, bone, soft tissue, or poorly perfused abscesses.

The blood–brain barrier (BBB) represents one of the most restrictive tissue barriers. Brain microvascular endothelial cells are connected by highly organized tight junctions and are ensheathed by pericytes, astrocytic end-feet, basement membrane, while specialized transport systems tightly regulate molecular exchange between the blood and the brain.^89^ Under physiological conditions, paracellular permeability is extremely limited, and most peptides display poor CNS penetration after systemic administration. For AMPs, BBB penetration depends not only on molecular size but also on charge, polarity, serum binding, and possible recognition by influx or efflux systems.^90,91^ Although neuroinflammation, meningitis, or systemic infection may transiently increase BBB permeability, this opening is heterogeneous, unpredictable, and often does not permit therapeutically relevant AMP concentrations in the CNS. Furthermore, cationic amphipathic AMPs may interact non-specifically with endothelial membranes or glycocalyx components, potentially increasing vascular toxicity. Therefore, treatment of CNS infections generally requires AMPs with demonstrated BBB penetration, local delivery strategies, or specialized formulations optimized for CNS delivery.^92^ BBB penetration has been demonstrated for some proline-rich AMPs. Apidaecin Api137, oncocin, drosocin, and drosocin Pro5Hyp all showed significant brain influx in mouse BBB transport experiments, confirming parenchymal penetration.^93^ However, this appears to be a peptide-specific property and should not be generalized to other AMPs.

Overall, systemic AMP efficacy should be evaluated using infection-site exposure and activity, not plasma concentration alone, because each tissue imposes distinct barriers to extravasation, diffusion, sequestration, intracellular access, and local inactivation.

### 3.4 Clearance and Elimination

After intravenous administration, AMP clearance determines the duration of systemic exposure. Small unbound peptides are usually cleared rapidly by renal filtration, whereas larger, hydrophobic peptides are more likely to undergo hepatic, splenic, or immune-mediated clearance (Figure 2).^86^

Renal filtration begins at the glomerulus, where blood crosses three major structures: the fenestrated endothelium, the glomerular basement membrane, and the podocyte filtration slit diaphragm.^94^ The fenestrated endothelium contains pores approximately 60 nm in diameter and is coated with a negatively charged glycocalyx composed mainly of heparan sulfate proteoglycans and sialoproteins. The glomerular basement membrane is a dense collagen IV/laminin matrix that is also highly anionic because of heparan sulfate content. Finally, the slit diaphragm, formed by nephrin and podocin, provides an effective filtration barrier with an approximate size cutoff of ∼40 nm.^94^ Most AMPs fall within the 1–10 kDa range and are therefore readily filtered through the glomerulus. Their cationic charge may further facilitate filtration through electrostatic interactions with negatively charged components.^95^ Importantly, glomerular filtration depends more strongly on hydrodynamic radius than on molecular weight alone.^95,96^ However, when AMPs are bound to plasma proteins such as albumin, the resulting complexes are largely excluded from glomerular filtration, thereby reducing renal clearance.^94,95^ Hence, peptide hydrophobicity and amphipathic organization indirectly modulate renal elimination through their effects on serum protein binding.

After filtration, AMPs enter the proximal tubule, where specialized cuboidal epithelial cells can reabsorb some AMPs very efficiently. The apical surface of these cells contains dense microvilli that maximize reabsorption. Here, a key receptor involved in AMP uptake is megalin, a multiligand endocytic receptor that recognizes ligands primarily through electrostatic interactions with positively charged residues.^97^ Cationic peptides bind avidly to megalin and are internalized through clathrin-mediated endocytosis.^98^ Following endosomal acidification, megalin releases the peptide because ligand binding is pH-sensitive. Megalin is then recycled to the apical membrane, whereas late endosomes fuse with lysosomes, where peptides are degraded by lysosomal cathepsins. Amino acid degradation products then exit through basolateral transporters. Thus, although peptides can be reabsorbed in the proximal tubule, they are generally degraded in lysosomes.^98^ Additionally, peptides may interact with cubilin, another large endocytic receptor that cooperates with megalin.^97^ AMPs may also bind directly to anionic components on the brush-border membrane, increasing their local concentration at the apical surface independently of megalin or cubilin. This may facilitate pinocytosis and intracellular accumulation.^99–101^

As a side effect, tubular reabsorption may also contribute to nephrotoxicity. The best clinical example involves polymyxins. After filtration and proximal tubular uptake, polymyxins accumulate within tubular epithelial cells and induce mitochondrial dysfunction, oxidative stress, lysosomal injury, membrane damage, inflammation, apoptosis, and necrosis. Clinically, this results in acute tubular injury characterized by elevated serum creatinine, reduced creatinine clearance, and, in severe cases, acute kidney injury.^102–104^ For AMPs, nephrotoxicity risk is expected to increase with high cationic charge, strong amphipathicity, elevated hydrophobicity, poor serum protein binding, slow intracellular degradation, or excessive proximal tubular accumulation. Membrane-active peptides are particularly problematic because they may damage mammalian membranes in addition to bacterial membranes when local concentrations become high enough.^104^ Strategies such as terminal modification, d-amino acid incorporation, constrained peptide structures, reduced non-specific hydrophobicity, or controlled serum-protein binding may reduce renal clearance or intracellular degradation.^95,105^ However, these same modifications may alter tissue distribution and shift elimination toward hepatic or immune-system clearance pathways rather than eliminating clearance altogether.

The liver constitutes a second major clearance route, especially for peptides that are too large, hydrophobic, strongly protein-bound, or formulation-associated to undergo rapid renal filtration (Figure 2).^106^ Hepatic sinusoids are lined by fenestrated liver sinusoidal endothelial cells and contain Kupffer cells, the resident macrophages of the liver.^107,108^ This organization facilitates efficient interaction between circulating macromolecules and hepatic clearance mechanisms. Liver sinusoidal endothelial cells express scavenger receptors and Fc receptors involved in clearance of macromolecules, immune complexes, pathogens, and circulating waste products, whereas Kupffer cells continuously sample the sinusoidal lumen and remove opsonized or particulate material. Hepatic peptide clearance may occur through multiple mechanisms. Hydrophobic or lipidated peptides may associate with albumin, lipoproteins, or membranes, increasing hepatic exposure as the liver plays a central role in lipoprotein and plasma-protein metabolism.^109^ Cationic peptides may additionally interact with anionic glycosaminoglycans or scavenger receptors on sinusoidal cells. Once internalized, peptides are delivered to endosomal and lysosomal compartments where they undergo proteolytic degradation.^110,111^ Larger or more hydrophobic peptide-derived metabolites may subsequently undergo biliary excretion, although this pathway is generally more relevant for modified peptides, conjugates, and nanoparticle formulations than for small unmodified AMPs, which preferentially undergo renal elimination.^109,112^ Hepatic clearance becomes especially important when AMP formulations are engineered to avoid rapid renal filtration. Strategies such as PEGylation and polymer conjugation, or formulations based on liposomes and nanoparticles, can prolong circulation time but often increase uptake by hepatic sinusoidal cells and macrophages. Consequently, strategies designed to reduce renal clearance may instead shift elimination toward the liver and spleen. This changes the pharmacokinetic and toxicity profile, potentially reducing rapid clearance and nephrotoxicity while increasing hepatic accumulation, macrophage uptake, complement activation, or inflammatory responses.^110,113^

Freely soluble peptides of approximately 2–5 kDa are are primarily cleared by renal filtration and proteolysis rather than phagocytosis. However, immune clearance becomes more important when AMPs aggregate, bind plasma proteins, associate with extracellular vesicles or bacterial debris, activate complement, or are incorporated into liposomes or nanoparticles. Under these conditions, plasma proteins form a “protein corona” containing complement proteins, immunoglobulins, fibrinogen, and other opsonins.^113,114^ Opsonized complexes are then recognized by Fc receptors, complement receptors, and scavenger receptors on macrophages, especially in the liver and spleen. This is a primary driver of mononuclear phagocyte system clearance for nanoscale drug carriers.^115^ Following internalization, complexes are trafficked to phagosomes, endosomes, and lysosomes, where peptides are degraded by acidic proteases and hydrolases.^116^ Repeated administration may further enhance immune-mediated clearance by inducing anti-drug antibodies or complement activation. This risk is higher for modified, aggregated, carrier-associated, or PEGylated peptides than for short unmodified AMPs. In these cases, immune recognition may shorten circulation half-life, increase hepatic and splenic uptake, and promote infusion-related inflammatory reactions.^117^

## 4 Inhaled Administration

Inhalation offers a targeted administration route for AMPs intended to treat respiratory infections because it can lead to high local concentrations in the airway lumen or epithelial lining fluid while limiting systemic exposure (Figure 3). This is the rationale behind inhaled polymyxins such as colistimethate sodium (Colobreathe) for chronic infection in cystic fibrosis,^118^ and behind newer inhaled AMP or peptidomimetic analogues such as murepavadin and SET-M33.^119^

**Figure 3:**
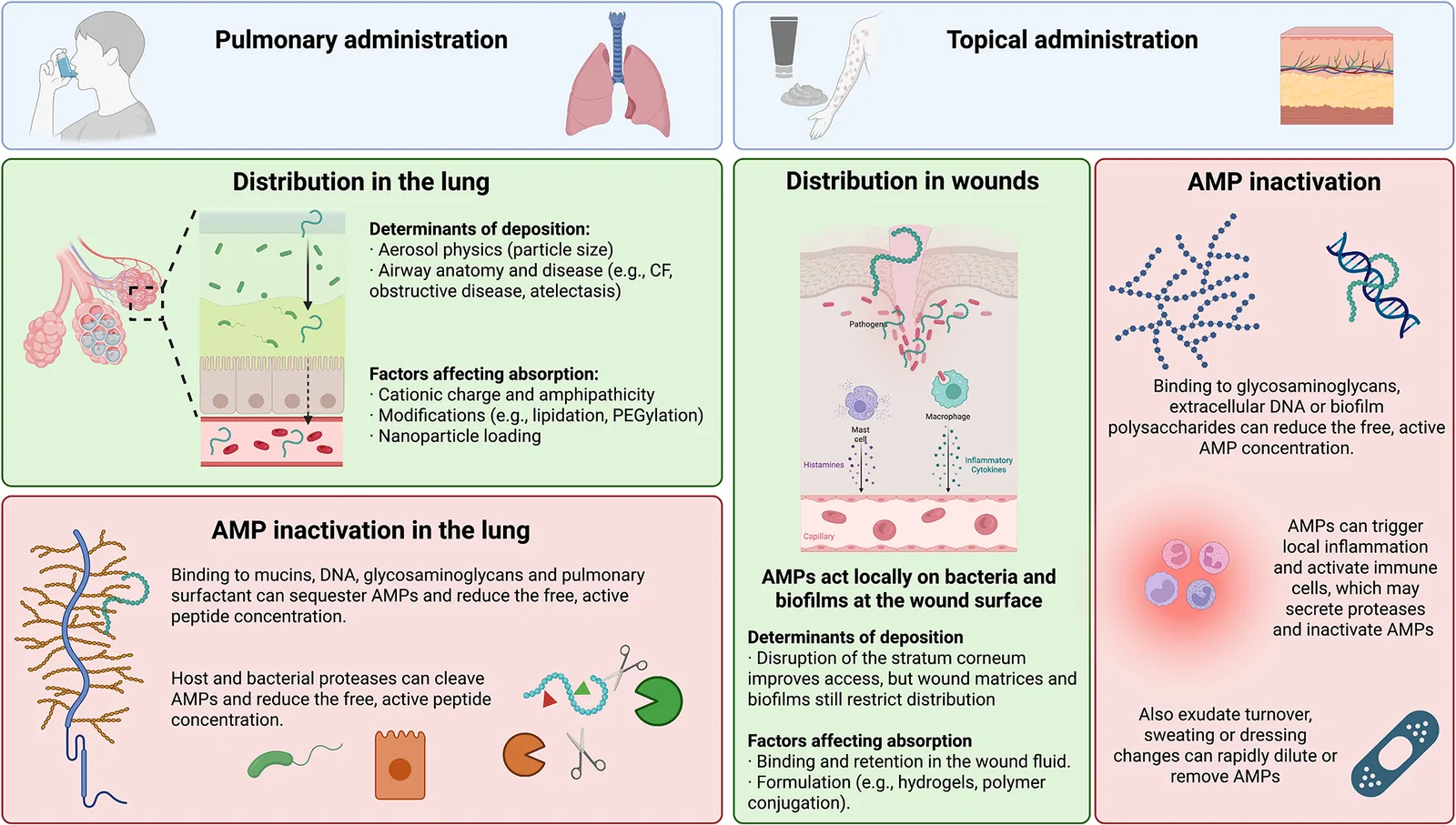
Pulmonary and topical delivery allow AMPs to achieve high local concentrations at the infection site while reducing systemic exposure. After inhalation, AMP distribution depends on aerosol deposition, airway anatomy and disease-related heterogeneity, including mucus obstruction and inflammation. In the lung, AMPs may be sequestered or inactivated by mucins, extracellular DNA, glycosaminoglycans and pulmonary surfactant, and can be degraded by host and bacterial proteases. After topical administration, AMPs distribute within the infected wound. Their efficacy is limited by local sequestration, proteolysis, dilution by exudate, dressing changes, and potential cytotoxic or inflammatory effects. In both routes, formulation and peptide chemistry determine the balance between local retention, free active peptide concentration, antimicrobial efficacy and toxicity. Created in https://BioRender.com.

AMPs can be delivered to the lung as nebulized solutions or suspensions, dry powder formulations delivered by inhalers or, less commonly, metered-dose aerosol systems.^120^ Nebulization is useful for peptides that are soluble and unstable during powder processing, whereas dry powders can improve portability and storage stability but require particle engineering. For deep-lung deposition, the aerosol must generally contain particles in the respirable aerodynamic range, commonly around 1–5 μm, as larger particles are more likely to deposit in the mouth and upper airways, whereas very small particles may be exhaled or reach alveoli inefficiently depending on breathing pattern and flow rate.^121^

For AMPs, formulation is a critical point. Linear cationic peptides may aggregate, adsorb to device surfaces, or lose activity during nebulization or drying. More stable designs, such as cyclic, branched, d-amino-acid-containing, or nanoparticle-associated peptides, are often better suited for inhalation because they resist proteolysis and may remain longer in the lung. For example, the branched AMP SET-M33 loaded onto dextran nanoparticles showed longer lung residence after aerosol delivery than free SET-M33 and retained activity for a longer time in a mouse pneumonia model.^119^

### 4.1 Distribution in the Lung

After inhalation, AMP distribution depends on aerosol physics, airway anatomy, and disease state.^122^ Infections in cystic fibrosis and bronchiectasis often involve mucus-rich conducting airways, whereas bacterial pneumonia may require delivery to distal bronchioles and alveolar spaces. Airway obstruction, mucus plugs, atelectasis, ventilation heterogeneity, and inflammation can all prevent uniform deposition.^123^ Therefore, even if the nominal inhaled dose is high, the peptide may not reach all infected regions.

Once deposited, the AMP partitions into the airway surface liquid, mucus, epithelial layers, biofilm matrix, and host cell membranes (Figure 3).^124^ Small soluble peptides may diffuse relatively well through hydrated airway surface liquid, but strongly cationic peptides can bind mucins, extracellular DNA, actin, glycosaminoglycans, and negatively charged bacterial or host polymers. This can increase local retention but reduce the freely diffusible antimicrobial fraction, which is especially relevant in cystic fibrosis sputum, where DNA, mucins, lipids, and proteases can influence AMP efficacy.

### 4.2 Absorption from the Lung to the Systemic Circulation

For treatment of airway infections, extensive systemic absorption is usually not desirable. The goal is to maintain high concentrations in the epithelial lining fluid while minimizing plasma exposure. Absorption depends on peptide size, charge, hydrophobicity, formulation, and epithelial integrity. Small, hydrophilic AMPs may be absorbed through the airway or alveolar epithelium more readily, especially if inflammation increases epithelial permeability. However, many AMPs are cationic and amphipathic, which promotes binding to mucus, surfactant, epithelial membranes, and extracellular matrix, slowing systemic absorption. Lipidation, PEGylation, or nanoparticle loading can further slow systemic absorption and extend lung residence, although these strategies may also reduce penetration through mucus or biofilm. Colistin is a useful example. Inhaled colistimethate can achieve high pulmonary exposure with lower systemic concentrations than intravenous administration, thereby improving local efficacy while reducing systemic nephrotoxicity risk.

### 4.3 Inactivation in the Lung

The lung is not simply a passive delivery compartment, and AMPs can be inactivated by several mechanisms. Electrostatic sequestration by negatively charged polymers such as mucins is a major problem (Figure 3). This lowers the concentration of free peptide available to interact with bacterial membranes. For example, LL-37 binding to glycosaminoglycan-rich fluids, including cystic fibrosis sputum, has been shown to inhibit antimicrobial activity despite high peptide levels.^125^ Pulmonary surfactant, which is rich in phospholipids and surfactant proteins, can also inactivate amphipathic or lipid-interacting peptides.^126^ Hydrophobic or lipidated AMPs may partition into this layer instead of bacterial membranes. Daptomycin is the clearest precedent: although it is a cyclic lipopeptide antibiotic rather than a classical AMP, it is inhibited by pulmonary surfactant because it interacts with surfactant lipids, reducing antibacterial activity in alveolar pneumonia.^126,127^ This illustrates a general risk for lipidated or strongly membrane-active peptide antibiotics. Proteolysis can also be substantial in infected lungs. In cystic fibrosis and bronchiectasis, chronic neutrophilic inflammation and bacterial protease production create a protease-rich environment that can shorten AMP residence time and generate inactive fragments.^128^ Neutrophil-rich airway secretions contain neutrophil elastase, proteinase 3, and cathepsin G, which together mediate extensive proteolysis: neutrophil elastase cleaves after small hydrophobic residues, proteinase 3 after aliphatic residues, and cathepsin G after aromatic and basic residues. Bacterial proteases can also contribute to AMP cleavage, ranging from broad degradation to selective cleavage of defined motifs.^129^ In CF-relevant lung pathogens, LasB and related metalloproteases preferentially cleave solvent-exposed regions enriched in hydrophobic or small neutral residues and can degrade LL-37, whereas other proteases have more defined specificities.^216^ For example, aureolysin is a zinc-dependent metalloprotease that preferentially cleaves peptide bonds adjacent to bulky hydrophobic residues, particularly isoleucine, leucine, phenylalanine, and tyrosine.^130,217^ Linear, flexible AMPs are especially vulnerable, whereas cyclic, disulfide-stabilized, d-amino-acid-containing, or branched peptides are more resistant. Finally, local physicochemical conditions also influence AMP activity. Ionic strength, divalent cations, pH, low oxygen tension, and high protein/lipid content can all modify AMP activity. Increased ionic strength and elevated concentrations of divalent cations such as Ca^2+^ and Mg^2+^ can shield electrostatic interactions between cationic AMPs and negatively charged bacterial surfaces, reducing peptide binding and membrane disruption.^131^ Changes in pH can alter AMP charge, conformation, and solubility, thereby affecting target recognition and antimicrobial potency.^132^ Low oxygen tension can reduce the efficacy of AMPs whose activity depends on oxidative stress pathways and can also induce bacterial physiological adaptations that increase AMP tolerance.^133^ In addition, many cationic AMPs are less active when electrostatic attraction to bacterial membranes is weakened or when host-derived anionic polymers outcompete bacterial membranes for AMP binding.

### 4.4 Removal and Clearance from the Lung

AMPs deposited in the conducting airways can be removed by mucociliary clearance,^134^ as ciliary transport continuously moves mucus-associated proteins and peptides toward the upper airway for expectoration, limiting their residence time and local antimicrobial activity. Small soluble peptides may also cross the epithelium and enter blood or lymph, after which they undergo renal filtration, hepatic uptake, or plasma proteolysis.^135^ Larger particles, aggregates, or nanoparticle formulations may be taken up by alveolar macrophages, especially if they are poorly soluble or opsonized. Peptides may also be degraded locally by epithelial cells, macrophages, and extracellular proteases. Hence, small soluble peptides tend to clear faster through absorption and proteolysis. Cationic peptides may be retained by mucus and extracellular polymers, whereas hydrophobic or lipidated peptides tend to partition into surfactant and membranes.^125,136^ Formulated AMPs, such as nanoparticle-associated peptides, may persist longer in the airways but can undergo clearance through macrophage-mediated uptake.

### 4.5 Potential Toxicity

The main toxicity risks are local respiratory reactions, epithelial injury, surfactant interference, inflammatory activation, and systemic toxicity if enough peptide is absorbed. Local airway reactions include cough, throat irritation, dysphonia, dyspnea, and bronchospasm. These are well documented for inhaled colistin products, where inhalation can induce coughing or bronchospasm, which is often managed with pretreatment with an inhaled β2-agonist.^137,138^

Mechanistically, toxicity is linked to the same properties that give AMPs antimicrobial activity.^139^ High cationic charge and amphipathicity favor bacterial membrane disruption, but at high local concentrations they can also perturb epithelial cell membranes, mitochondrial membranes, or surfactant films. Excessive hydrophobicity increases membrane insertion and cytotoxicity, whereas strong positive charge can promote mucus binding, inflammatory complex formation, and interaction with host glycosaminoglycans. For nanoparticle or carrier-based formulations, toxicity can shift from peptide-mediated membrane damage toward particle-associated inflammation, excessive macrophage uptake, complement activation, or impaired mucociliary clearance.^119^ However, formulation can also reduce toxicity by controlling release and lowering the local concentration of free peptide. The SET-M33 dextran nanoparticle study is a relevant example in which nanoparticle association improved lung residence while demonstrating acceptable cytotoxicity in preclinical assays.^119^

## 5 Topical Administration

Topical administration is one of the most extensively studied routes for AMP therapy because it avoids most systemic barriers such as serum binding, renal clearance, and hepatic uptake. The goal is to generate a high local AMP concentration at the infected surface while limiting systemic exposure (Figure 3). This route is most relevant for infected wounds, burns, diabetic foot ulcers, venous ulcers, catheter exit sites, acne, atopic dermatitis, seborrheic dermatitis, and some mucosal infections.^140,141^ In these cases, AMPs can be applied as creams, ointments, gels, sprays, hydrogel dressings, impregnated wound dressings, catheter coatings, or controlled-release biomaterials. Some well-studied clinical candidates include pexiganan, a synthetic 22-amino-acid magainin analogue tested as a topical cream for mildly infected diabetic foot ulcers;^142,143^ omiganan, a short cationic indolicidin-derived peptide developed as a topical gel for skin and catheter-associated indications;^144–146^ and LL-37, which has been evaluated topically in chronic leg ulcers.^147,148^

### 5.1 Distribution and Absorption

After application, the AMP distributes from the vehicle into the local environment, including stratum corneum, wound fluid, mucus, biofilm matrix, necrotic tissue, or the catheter–skin interface. For intact skin, penetration is usually limited by the stratum corneum. This can be advantageous if the target is superficial colonization, because the peptide remains local. However, it becomes limiting if bacteria are in deeper epidermal or dermal layers. For open wounds and ulcers, the barrier is very different.^149^ The stratum corneum is disrupted, so AMPs can access wound fluid, exposed extracellular matrix, immune cells, and biofilms more directly. However, diffusion is heterogeneous. Chronic wounds often contain necrotic tissue, fibrin, extracellular DNA, serum proteins, proteases, bacterial products, and biofilm matrix, and these components can bind or degrade AMPs before the peptides can eliminate all bacteria.^150–152^ For catheter exit sites or device coatings, the objective is local retention at the skin-device interface, where bacteria first adhere and form biofilms. Omiganan is a useful example because it was developed as a topical cationic peptide for local prevention of catheter-associated infections and has also been explored in inflammatory skin diseases.^144,153^

Systemic absorption after topical AMP application is usually low, especially on intact skin, because most AMPs are relatively large, polar, and cationic compared with classical small-molecule drugs.^154^ However, absorption can increase when the epithelial barrier is disrupted, as in burns, ulcers, large wounds, inflamed skin, or mucosal surfaces. Small linear peptides may diffuse more readily through wound fluid and superficial tissue but are also more vulnerable to proteolysis. Furthermore, highly cationic peptides bind strongly to anionic tissue components such as glycosaminoglycans, extracellular DNA, acidic phospholipids, and bacterial cell envelopes, increasing local retention but reducing deeper penetration and free active concentration. On the other hand, hydrophobic or lipidated peptides may partition into membranes, sebum, necrotic lipids, or dressing materials. This may improve residence time but can increase host-cell toxicity. PEGylated, polymer-conjugated, nanoparticle-loaded, or hydrogel-entrapped AMPs generally show lower systemic absorption and longer local residence, but they may penetrate biofilms or dense tissue less efficiently unless release is well controlled.^149,155,156^

Although topical delivery removes the need for systemic absorption and limits systemic exposure, some of the barriers discussed so far still apply, as the peptide must remain free, intact, and diffusible within the infected microenvironment.

### 5.2 Inactivation at the Topical Site

Topical microenvironments can strongly inactivate AMPs. The main mechanisms are sequestration, proteolysis, and physicochemical inhibition. Many AMPs are cationic because they need to bind bacterial membranes.^150,152,157^ The same property promotes binding to host and microbial anionic polymers, including extracellular DNA, mucins, glycosaminoglycans, alginate, teichoic acids, LPS, and biofilm polysaccharides. This can be particularly problematic in pus-rich wounds and chronic ulcers, where DNA and cell debris can act as a large binding sink.^87,152^ Wounds and inflamed skin contain host and bacterial proteases. As noted in the context of lung infections, neutrophil elastase, cathepsin G, proteinase 3, bacterial metalloproteases, and staphylococcal or pseudomonal proteases can degrade susceptible AMPs. Again, linear, flexible α-helical peptides are usually more vulnerable than cyclic, d-amino-acid-containing, disulfide-stabilized, or otherwise constrained peptides. This is one reason why formulation strategies often aim to protect AMPs from proteases while releasing them gradually into the wound. Recent reviews of AMP wound delivery emphasize that antimicrobial effects, cytotoxicity, inflammation, and wound-healing outcomes depend strongly on formulation and release kinetics.^149,158^

### 5.3 Local Clearance

Topically applied AMPs are cleared by several routes including (i) physical removal through wound exudate, dressing changes, washing, sweating, or desquamation, (ii) proteolytic degradation into inactive fragments at the wound or skin surface, and (iii) cellular uptake by keratinocytes, fibroblasts, neutrophils, or macrophages. In chronic wounds in particular, exudate turnover can be high, rapidly diluting or removing deposited peptides.^152^ A peptide with excellent in vitro potency may be rapidly diluted or removed unless the formulation maintains sustained local release. Hydrogels, nanofibers, wound dressings, and polymer matrices are therefore advantageous because they can maintain a local depot and reduce repeated dosing.^155,156,159^

### 5.4 Potential Toxicity

Topical AMPs can cause local and, less commonly, systemic toxicity. Cationic amphipathic peptides can activate mast cells and keratinocytes and engage innate immune pathways (Figure 3).^160,161^ Mild inflammation may aid wound repair, but excessive activation can delay healing or worsen dermatitis. For example, high hydrophobicity, high amphipathicity, lipidation, and high local concentration increase the risk of keratinocyte, fibroblast, endothelial-cell, or erythrocyte membrane damage.^162^ This is mechanistically linked to the same membrane-disruptive properties that confer antimicrobial activity. If the AMP damages fibroblasts, keratinocytes, endothelial cells, or extracellular matrix organization, it may impair recovery. This is particularly important for diabetic ulcers and venous ulcers, where tissue repair is already compromised.^163^ Even if a single application causes no detectable adverse effects, repeated topical exposure to modified peptides, carrier systems, or formulation excipients may produce local hypersensitivity, although the risk depends strongly on peptide sequence, formulation, and exposure duration. Finally, broad-spectrum AMPs may reduce pathogen load but also alter commensal skin microbiota.^164,165^ This can be useful in dysbiosis-associated conditions but may also disturb barrier homeostasis.

The risk profile is therefore tightly linked to AMP chemistry. A peptide with very high cationic charge may bind bacteria well but also bind host glycosaminoglycans and wound debris. A very hydrophobic peptide may disrupt membranes efficiently but may be more cytotoxic. A highly protease-resistant peptide may last longer but may also accumulate locally. Conversely, a rapidly degraded peptide may be safer but may fail to maintain effective exposure.

## 6 Clinical Translation: Lessons from Selected AMP Candidates

Clinical outcomes are seldom determined by simple physicochemical properties alone. Table 1 illustrates how strong local antimicrobial activity may still translate into modest clinical benefit, a narrow dose–response profile, route-dependent toxicity, or endpoints strongly influenced by wound care and tissue repair. Such cases emphasize the need to align route of administration, formulation, biological matrix, dose, host response, and endpoint selection before an AMP advances to confirmatory testing. A solid development strategy requires a target product profile that defines a clear connection between peptide exposure and microbial killing, leading to an outcome that is both mechanistically linked to AMP activity and clinically significant.^142–148,166^

**Table 1:** Translational Outcomes for Selected AMP Clinical Studies

candidate	indication, outcome, and translational barrier
pexiganan	indication: topical cream for mildly infected diabetic foot ulcers
	outcome: in two randomized controlled trials, study 303 failed to demonstrate equivalence, whereas study 304 and the pooled analysis showed clinical improvement rates similar to oral ofloxacin (85–90%). Pexiganan was not approved by the US FDA^142^
	translational barrier: broad in vitro activity against diabetic-foot isolates did not establish added clinical benefit beyond an active comparator and standard wound care, while the heterogeneous ulcer environment introduced additional variability.^142,201^
omiganan	indication: topical gel for inflammatory dermatoses
	outcome: in randomized dermatology studies, omiganan altered the microbiome in atopic dermatitis with only modest clinical effects of uncertain relevance, and showed no significant clinical efficacy in facial seborrheic dermatitis.^145,153^
	translational barrier: a strong microbiological signal does not guarantee improvement in a host-dominated endpoint. Vehicle effects, baseline microbiota, inflammation, and endpoint selection can dominate the observed response
LL-37	indication: topical treatment for hard-to-heal venous leg ulcers
	outcome: a 34-participant first-in-human trial showed faster healing at 0.5 mg/mL and a weaker signal at 1.6 mg/mL, but no benefit at 3.2 mg/mL. A later 148-participant multicenter phase IIb trial confirmed safety but found no significant healing improvement in the full study population.^147,148^
	translational barrier: antimicrobial, immunomodulatory, cytotoxic, and wound-repair effects may have different dose optima. Dose-ranging should therefore measure bacterial burden, inflammation, tissue tolerance, and healing rather than assume that more peptide is better
iseganan	indication: oral rinse for prevention of radiotherapy- or chemotherapy-associated oral mucositis
	outcome: two large, randomized phase III trials found no significant reduction in ulcerative mucositis or its clinical sequelae versus placebo. In the radiotherapy trial, both iseganan-plus-care and placebo-plus-care outperformed standard oral care alone for some outcomes.^202,203^
	translational barrier: a broad-spectrum antimicrobial can fail when host tissue injury, rather than microbial burden, is the dominant pathology
murepavadin	indication: intravenous treatment of hospital-acquired or ventilator-associated bacterial pneumonia
	outcome: the intravenous phase III study was halted in 2019 after acute kidney injury was observed in 56% of murepavadin-treated participants in the ventilator-associated bacterial pneumonia study. Earlier phase I studies had supported tolerability and characterized pharmacokinetics, including after a single dose in participants with renal impairment.^204–206^
	translational barrier: pathogen selectivity does not guarantee organ selectivity. Repeated-dose renal exposure and toxicity must be modelled under the intended systemic regimen
surotomycin	indication: oral, minimally absorbed cyclic lipopeptide for *Clostridioides difficile* infection
	outcome: two parallel phase III trials produced discordant primary results: one failed to demonstrate non-inferiority to vancomycin, whereas the other met non-inferiority for end-of-treatment clinical response but not superiority for sustained response.^207,208^
	translational barrier: promising phase II activity and a microbiome-sparing rationale were insufficient. Replication across clinical trials, comparator performance, strain mix, recurrence, and sustained response can determine value more strongly than initial cure or in vitro selectivity
daptomycin	indication: intravenous daptomycin for complicated skin and skin-structure infection, bacteremia, and right-sided endocarditis
	outcome: daptomycin was non-inferior to standard therapy for bacteremia and right-sided endocarditis. It failed non-inferiority in severe community-acquired pneumonia, and pulmonary surfactant was shown to inhibit its antibacterial activity.^126,209,210^
	translational barrier: a peptide antibiotic can succeed when free exposure, pathogen spectrum, and indication align, yet an organ-specific biological matrix can create an absolute barrier. Activity should not be extrapolated across compartments
polymyxins	indication: intravenous colistin or polymyxin B, and inhaled colistin, for severe multidrug-resistant Gram-negative infections
	outcome: these agents remain important last-line therapies, but interpatient pharmacokinetic variability, a narrow therapeutic window, and nephrotoxicity constrain systemic dosing. Inhaled colistin can cause bronchoconstriction, particularly in patients with coexisting cystic fibrosis and asthma.^137,211–213^
	translational barrier: high unmet need can sustain clinical use despite a narrow therapeutic index, but the acceptable exposure-safety window and formulation are route-specific. Clinical utility is not evidence that a scaffold is fully optimized

No single ex vivo system has been validated as a surrogate for clinical efficacy. Even so, a staged set of route-matched models is far more informative than broth MIC measurements in isolation (Figure 4). Evaluation of ral candidates should combine MIC determination with simulated stability in gastric and intestinal fluids, epithelial permeability assays and Ussing chambers. For intravenous candidates, antimicrobial activity and unbound peptide concentrations can be measured complementarily in plasma, whole blood, or tissue homogenates, and endothelial or organ-specific models can be used to address barrier transport and toxicity. Inhaled peptides require assessment of antimicrobial activity in artificial or patient-derived sputum, mucus, airway surface liquid, pulmonary surfactant, and cytotoxicity in differentiated air-liquid interface cultures. Finally, evaluation of topical candidates can include activity in wound fluid or exudate, retention in collagen, fibrin matrices, or infected skin equivalents, and analysis in dynamic biofilm systems.^28,124,135,149,151,152,167^ These models capture selected translational constraints, but should not be used as complete surrogates for animal models as they cannot fully reproduce vascular delivery, immune-cell recruitment, tissue repair, repeated exposure, or clearance at the organ level.

**Figure 4:**
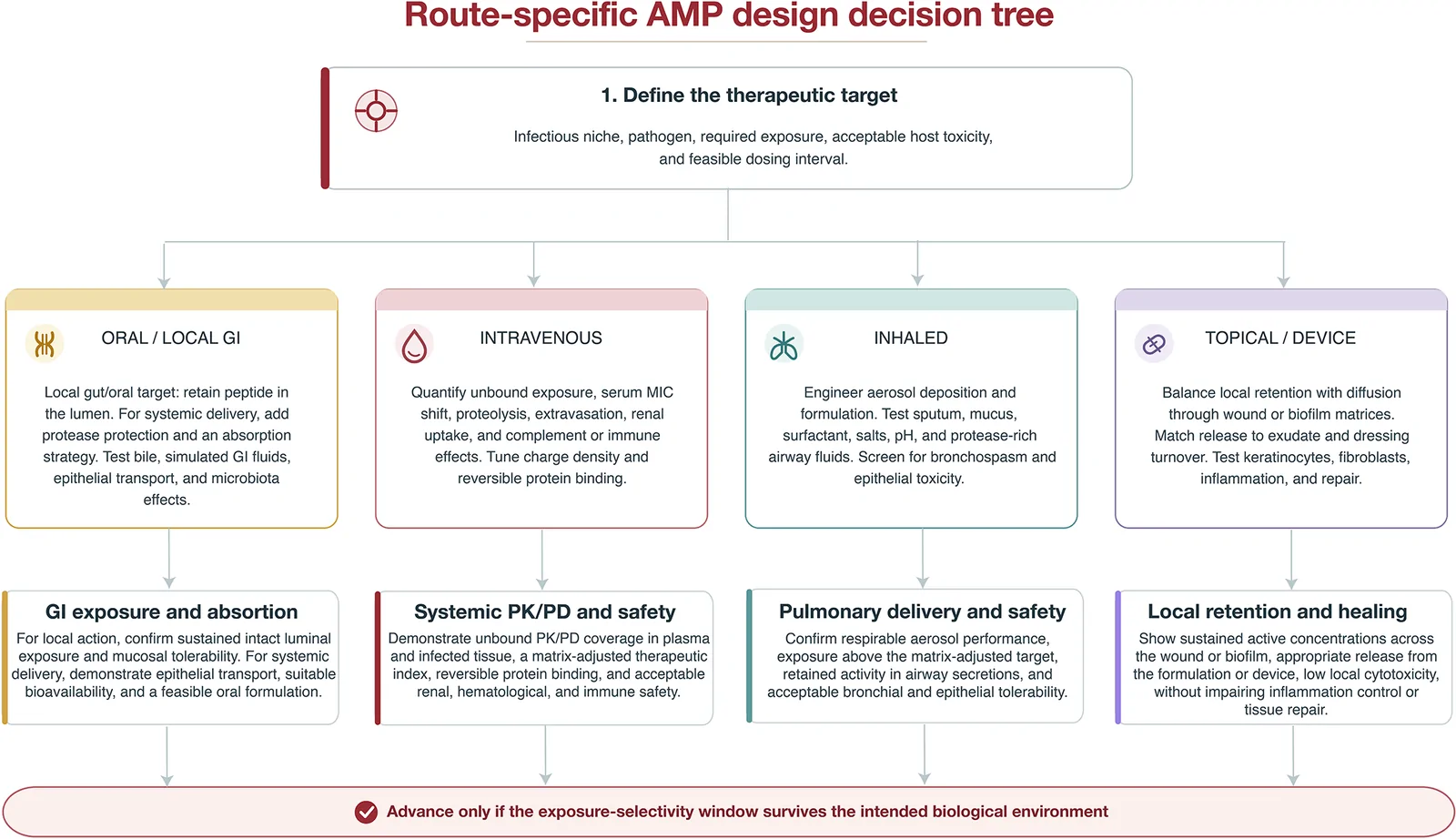
Route-specific decision framework for antimicrobial peptide development. The intended infectious niche, target pathogen, required exposure, acceptable host toxicity and feasible dosing interval are first defined to guide selection of the administration route. Each branch summarizes the principal design and testing priorities for oral or local gastrointestinal, intravenous, inhaled, and topical or device-based delivery. Route-specific progression criteria integrate gastrointestinal exposure or absorption, systemic unbound pharmacokinetic-pharmacodynamic coverage and safety, pulmonary deposition and tolerability, or local retention, release and tissue repair. Candidates should advance only when acceptable activity and exposure are maintained within a suitable selectivity window under the biological conditions relevant to the intended route.

Animal models should be chosen with the same attention to route and intended mechanism. In systemic studies, neutropenic murine thigh, peritonitis, and disseminated-infection models can support estimation of direct antibacterial PK/PD by reducing neutrophil-dependent bacterial clearance.^168,169^ However, immunocompetent cohorts should be included when host-defense or immunomodulatory effects contribute to the proposed mechanism.^170^ Inhaled candidates should be evalutated in indication-matched models, ranging from acute intratracheal or aerosol pneumonia models to chronic, mucus-rich, or cystic-fibrosis-relevant settings and, ideally, regional deposition in the lung should be measured, not inferred from the nominal dose. Topical compounds can be assessed in excisional, burn, diabetic, or biofilm-infected wound models but porcine skin or wound systems should also be considered, because rodent skin heals largely through contraction and differs substantially from human skin in thickness and repair dynamics. Device-related indications, require catheter- or implant-associated biofilm models, whereas abscess indications call for structured, poorly perfused lesions rather than planktonic infection challenges.^78,147,148^

Predictive power nonetheless remains limited. Many rodent infection studies model acute disease using large inocula, short observation periods, young animals without relevant comorbidities, and treatment is often initiated shortly after bacterial challenge.^171,172^ Human cystic-fibrosis lungs, chronic wounds, and device-associated biofilms present a different problem: established microbial communities, uneven drug exposure, comorbid disease, and repeated prior treatment. If candidates are not properly tested, there is a high risk of translational failure.^173,174^ Pexiganan, omiganan, and LL-37 each produced encouraging preclinical or early clinical signals, but none produced a strong and reproduible clinical benefit (Table 1). The underlying reasons varied, but included limited superiority over standard care, strong dependence on endpoint choice, and failure to convert local antimicrobial activity into meaningful clinical benefit.^142,145,147,148,166^ Together, these examples show how favorable model results may overstate the magnitude or reliability of the eventual clinical effect. A rigorous development should therefore seek agreement across human-derived matrices, complementary in vivo models that test different translational assumptions, direct measurements of unbound PK/PD, and an endpoint that can be transferred into the clinical protocol.

## 7 Design Strategies to Overcome Administration Barriers in AMP Therapy

The development of AMPs as therapeutic agents requires more than improving the antimicrobial activity in vitro. Most AMPs kill bacteria efficiently under optimized laboratory conditions but fail when used in relevant biological environments. Therefore, AMP design should incorporate an integrated pharmacological objective: maintaining a sufficient concentration of intact, active, non-toxic peptide at the infection site for long enough to eliminate the pathogen. This objective requires simultaneous optimization of stability, distribution, tissue penetration, selectivity, formulation, and excretion.^11,13^

A first design principle is that AMPs must be designed for the intended administration route. An AMP optimized for topical administration in wounds does not necessarily require the same properties as one designed for the treatment of sepsis, or a peptide intended for inhaled treatment of pneumonia. For oral systemic therapy, the main barriers are gastrointestinal proteolysis, bile sequestration, and ineffective epithelial absorption. In this context, unmodified AMPs are generally inadequate unless they are designed for the treatment of local infections in the intestine or oral cavity. Oral administration for systemic purposes typically requires formulation enhancements, such as protease protection, permeation enhancers, receptor-targeted carriers, nanoparticles, or prodrug strategies.^12^ On the other hand, AMPs intended for intravenous administration must be optimized for plasma stability, controlled serum protein binding, efficient tissue extravasation, minimal renal toxicity, and limited immune clearance. Similarly, topical and inhaled AMPs should prioritize local retention and activity within the target environment (e.g., mucus, wound exudate, or biofilm matrices), with low local cytotoxicity.^149^

Resistance to proteases is one of the most fundamental and broadly applicable design requirements. Linear AMPs with free termini and exposed cleavage sites are vulnerable to proteases, including gastrointestinal, plasma, and neutrophil proteases, bacterial proteases, and lysosomal enzymes in intracellular infections.^69^ Several modifications can enhance stability, including N-terminal acetylation, C-terminal amidation, substitution with d-amino acids, incorporation of non-natural residues, cyclization, stapled peptides, and conformational constraints such as disulfide or lactam bonds.^72^ These strategies reduce access to proteases and can stabilize active conformations. However, resistance to proteases should not be maximized without considering toxicity and elimination. A highly active but poorly degradable peptide may persist longer in host tissues, increasing the risk of cytotoxicity, whereas a moderately degradable peptide can retain sufficient antimicrobial activity while limiting tissue accumulation. Hence, the objective is not absolute protease resistance, but controlled stability within the required therapeutic window.^13^

Controlled stability should be defined by how long an intact, biologically active peptide persists within the matrix and compartment of interest, rather than merely measuring how quickly the parent peptide is degraded in buffer. Although universal target ranges have not been validated, published pharmacokinetic and formulation studies provide route-specific precedents for defining provisional exposure windows. While early gastrointestinal stability and release may be assessed over the first hours after local oral delivery, rapidly cleared intravenous peptides require characterization of early unbound exposure and the relevant PK/PD index.^175,176^ Inhaled agents should maintain measurable active concentrations in epithelial lining fluid over several hours, whereas topical formulations should preserve antimicrobial activity across the intended interval between applications or dressing changes.^177–179^ Depending on the compound and formulation, relevant exposure may extend well beyond these initial windows. Hence, the exposure required should be established from matrix-adjusted antibacterial potency, concentration- and time-dependent killing, unbound AUC/MIC or another appropriate PK/PD index, and the intended dosing interval.^13,25,69,86,95^ Documented examples in which excessive peptide stability alone has led to clinical failure are uncommon. However, the similar murine efficacy of protease-susceptible L-DFT561 and protease-resistant D-DFT561 suggests that additional stabilization may offer limited benefit once sufficient exposure has already been achieved.^180^ The converse issue is prolonged persistence. Renal accumulation of polymyxins and immune-mediated clearance of long-circulating modified constructs show that persistence must be considered together with tissue distribution, local peptide burden, immunogenicity, and safety during repeated dosing.^102,103,117,181^

Toxicity reduction must be integrated from the earliest stages of AMP design. Hemolysis, epithelial injury, nephrotoxicity, bronchospasm, complement activation, mast cell activation, or impaired wound healing are all plausible side effects of AMP treatment.^182^ Nephrotoxicity is especially relevant for small, cationic AMPs that undergo glomerular filtration and accumulate in proximal tubular cells.^183^ Reducing non-specific hydrophobicity, preventing excessive positive charge, and controlling binding to serum proteins can reduce the risk. For inhaled peptides, interaction with lining fluids and local irritation must be considered,^184^ whereas for topical AMPs, toxicity to keratinocytes and fibroblasts is a particular concern, as these cells are essential for tissue repair.^160,161^

Hence, a central strategy is to optimize the balance between charge and hydrophobicity rather than simply increasing both. A positive charge favors binding to the bacterial envelope, particularly LPS, teichoic acids, and negatively charged phospholipids, but excessive cationicity can increase non-productive binding to albumin, AGP, glycosaminoglycans, extracellular DNA, mucus, biofilm polymers, and renal brush-border membranes. Similarly, hydrophobicity increases membrane insertion, but excessive hydrophobicity also increases binding to serum proteins, partition into lipoproteins, hemolysis, epithelial toxicity, binding to surfactants, and hepatic uptake and clearance.^167^ The most useful AMPs are generally those that have an intermediate physicochemical range with sufficient amphipathic character to disrupt bacterial membranes but hydrophobicity and charge low enough to avoid sequestration in host matrices or damage to host cell membranes. Hence, the rational adjustment of hydrophobic moment, charge distribution, aromatic residue placement, and conformational flexibility is much more informative than optimizing only the net charge or hydrophobicity.^162,167,185^

For short, linear AMPs, a net charge of roughly +2 to +6 at pH 7.4 is a sensible starting point, though not a universal optimum. For peptides targeting Gram-positive bacteria, values toward the lower end may support systemic exposure by reducing nonspecific binding and host-cell toxicity. In contrast, Gram-negative activity can impose different constraints, requiring either stronger electrostatic engagement with the outer membrane or a more favorable spatial display of cationic residues. Net charge alone is therefore insufficient and should be considered alongside peptide length, charge density, Lys/Arg clustering, and the three-dimensional presentation of those residues.^186^ In a systematic series of database-derived peptides, analogues containing three to four lysines lost efficacy in neutropenic mice, whereas less cationic variants remained active despite similar in vitro potency.^180^ The observation is specific to that sequence series, but it nonetheless cautions against treating maximal positive charge as a general design objective. Hydrophobic moment and mean hydrophobicity, including GRAVY, remain useful for ranking analogues within a congeneric series. Apparent logP or logD may also help reveal the consequences of lipidation and nonspecific partitioning. These descriptors, however, do not provide a portable numerical threshold for hemolysis across peptides that differ in length, conformation, or charge state.^167,182,183^ An exposure-based boundary is more informative. Candidates should be rejected or redesigned when hemolysis or mammalian-cell toxicity occurs close to the therapeutic concentration. During early lead selection, a matrix-adjusted selectivity index above one order of magnitude can serve as a minimum screening benchmark. Similarly, a reproducible serum- or matrix-induced MIC increase of at least fourfold should prompt mechanistic studies of binding, sequestration, and formulation rather than automatic dose escalation.

Another strategy is to control binding to host proteins and matrices. Binding to serum proteins is normally considered negative because it lowers the concentration of the free, active peptide, but it can also prolong systemic circulation, protect the peptide from proteolysis, and reduce renal filtration. Albumin-binding motifs, lipidation, PEGylation, or conjugation can be useful when prolonged systemic exposure is required.^11,13^ The same principle can be applied to local applications. Binding to mucus, extracellular DNA, or other polymers can retain the peptide at the infection site or inactivate it by sequestration. Hence, AMP design should favor reversible, productive binding over irreversible sequestration by host components. Assays designed to evaluate binding to relevant biological matrices, such as serum, sputum, wound fluid, pus, or biofilm models, are essential for identifying peptides that retain activity in these environments rather than relying solely on minimum inhibitory concentration (MIC) in broth assays.^187,188^

For intravenous AMPs, no universal optimum exists for binding to albumin or α1-acid glycoprotein. The unbound fraction reflects more than affinity alone. It also depends on protein abundance, binding stoichiometry, site heterogeneity, competing ligands, and the kinetics of association and dissociation.^189,190^ Human albumin circulates at approximately 0.5–0.75 mM. In a simple 1:1 model where albumin is present in large excess, a *K*_d_ value of 1–10 μM would therefore predict that over 98% of the peptide is bound at equilibrium. Such affinity may still be considered moderate at the molecular level, yet its effect on peptide availability becomes substantial when the binding partner is present at sub-millimolar concentrations. Within this simplified framework, partial albumin binding requires a *K*_d_ value of roughly the same order as the albumin concentration. AGP is far less abundant, although its levels can rise markedly during inflammation, so partial binding may occur at lower micromolar affinities under typical physiological conditions.^44,45,50,55^ These calculations are useful for screening, but they cannot replace direct measurement. More informative development parameters include the concentration-dependent unbound fraction, dissociation rate and serum-induced change in antibacterial activity. The practical objective is straightforward: maintain sufficient free exposure while avoiding binding that is effectively irreversible over the relevant dosing interval. A reproducible serum-induced MIC increase of no more than approximately fourfold may provide an early operational benchmark, although it should be assessed by direct peptide quantification. Extensive protein binding may still support efficacy when dissociation is rapid and total exposure creates an adequate unbound reservoir, as illustrated by several long-acting peptide therapeutics. For AMPs, however, this behavior should be shown experimentally rather than inferred from total plasma half-life alone.^43,48^

Formulation is often the most effective way to bypass route-specific delivery barriers.^191–193^ Liposome encapsulation, polymeric nanoparticles, hydrogels, or nanofibers can protect AMPs from degradation, control release kinetics, reduce local toxicity, and improve residence at the infection site. For inhaled administration, aerosol particle engineering can adjust aerodynamic size to enhance pulmonary deposition and minimize systemic absorption. In topical administration, hydrogels maintain sustained local exposure even in the presence of wound exudate turnover. For oral administration, protective carriers can shield peptides from gastrointestinal proteases and enhance intestinal epithelial absorption. For intravenous therapy, nanoparticles and conjugates can extend peptide half-life, although they may also shift clearance towards the liver, spleen, or macrophages. Hence, formulation should be considered an integral component of the AMP therapeutic entity.

Penetration into hard-to-reach infection sites requires additional design criteria. Biofilms demand peptides that resist capture by the extracellular matrix of polysaccharides, DNA, and proteins, while retaining activity against slow-growing bacteria.^166,181,187^ Intracellular infections require efficient cellular uptake, endosomal escape, and activity within the relevant subcellular compartment, without excessive toxicity to the host cell. Abscesses, necrotic tissue, and purulent lesions require protease resistance and reduced binding to cellular debris. CNS infections require specialized strategies, as the BBB restricts entry of most peptides.^194^ Hence, formulation should be considered an integral com-ponent of the AMP therapeutic entity, not merely accessory.

AMP optimization should use assays that match expected clinical use. MIC values in broth are standard, but they are insufficient to predict therapeutic outcome. Candidate AMPs should be tested in the biological matrices relevant to the intended route: serum or plasma for systemic use, gastric or intestinal fluids for oral administration, sputum or mucus-like media for inhalation, suitable wound matrices for topical administration, and cell-based models for intracellular infections. Pharmacokinetic and pharmacodynamic studies should quantify the total peptide, free active fraction, active fragments, protein-bound fractions, and toxicity thresholds. Combination therapy with antibiotics, antibiofilm compounds, protease inhibitors, or immunomodulatory treatments may yield better outcomes than AMP monotherapy.

Here, contrasting AMPs with other successful peptide therapeutics helps clarify the central problem. As an illustrative example, semaglutide is a modified peptide that engages the cell-surface GLP-1 receptor with affinity in the low nanomolar range. Its sequence, lipidation, albumin binding, and resistance to proteolysis were all deliberately engineered to prolong systemic exposure, producing an intravenous plasma half-life of 46 h in minipigs.^195^ Most membrane-active AMPs operate in a very different pharmacological regime. Rather than binding a receptor that can initiate amplified signaling at low occupancy, they typically require micromolar concentrations to disrupt bacterial membranes. Effective concentrations are therefore several orders of magnitude higher. At the same time, selectivity must be preserved in the presence of host interfaces that may bind, sequester, internalize, or be damaged by AMPs. Proximal-tubule uptake is a prominent example, but erythrocytes, vascular and epithelial surfaces, immune cells, plasma components, and injured tissues may also reduce free exposure or compress the therapeutic window.^94,97–104,181–183^ Protease resistance is often achievable. The harder medicinal-chemistry task is to expand the therapeutic index without sacrificing sufficient exposure at the infected site. This exposure-selectivity window should therefore act as a central integrated optimization criterion, rather than maximal stability or broth potency considered separately.

Artificial intelligence and machine learning may render this multi-parameter problem more manageable, provided that the training objective reflects the intended route and experimental setting.^196^ Broth MIC alone is not a suitable target. Models should be trained on endpoints including matrix-adjusted activity, hemolysis, host-cell toxicity, proteolytic stability, serum binding or unbound fraction, aggregation, tissue penetration, and formulation compatibility.^197,198^ Yet, these measurements are highly protocol dependent. For this reason, assay conditions, biological matrix, species, peptide concentration, and exposure duration should be encoded together with sequence-derived descriptors. Conditional generative models could then prioritize candidates for a defined pathogen, route, and product profile, while active-learning approaches identify the next experiments most likely to reduce uncertainty. Multiobjective or Pareto optimization is particularly appropriate because changes in charge, hydrophobicity, or stability often improve one property at the expense of another.^199,200^ For such approaches, data quality and coverage remain the limiting factors. Current datasets are small, heterogeneous, biased towards short cationic peptides, and frequently omit negative findings on toxicity, pharmacokinetics, matrix-dependent activity, and unsuccessful optimization steps. Inconsistent assay conditions and leakage between homologous sequences can further exaggerate apparent model performance. Scaffold- or family-aware data splits, prospective evaluation in route-relevant matrices, and blinded testing in an external laboratory are therefore required before an AI-derived score can reasonably be treated as translational evidence.

Overall, the most promising AMP therapies are not simply those with the most potent sequences against pathogens in vitro. Effective translation requires peptides or formulations tailored to a specific clinical indication, infectious niche, and therapeutic context. Successful design demands balancing antimicrobial potency with proteolytic stability, controlled binding to host proteins and structures, efficient tissue penetration, low toxicity, and appropriate clearance. An integrated approach that addresses these parameters simultaneously is essential to bring AMP-based therapies into clinical practice.

## Notes

The Systems Biology of Infection Laboratory, Department of Biochemistry and Molecular Biology, Biosciences Faculty, Universitat Autònoma de Barcelona, Cerdanyola del Vallès, Barcelona 08193, Spain

## Abbreviations

AGP: α1-acid glycoprotein
AMP: antimicrobial peptide
ASBT: apical sodium-dependent bile acid transporter
BBB: blood–brain barrier
CF: cystic fibrosis
CNS: central nervous system
Dab: diaminobutyric acid
DPP4: dipeptidyl peptidase 4
FA: fatty acid-binding site
FcRn: neonatal Fc receptor
FDA: U.S. Food and Drug Administration
HDL: high-density lipoprotein
HNP-1: human neutrophil peptide 1
ITC: isothermal titration calorimetry
LDL: low-density lipoprotein
log*P*: octanol–water partition coefficient
LPS: lipopolysaccharide
MIC: minimum inhibitory concentration
NMR: nuclear magnetic resonance
PEG: polyethylene glycol
VLDL: very-low-density lipoprotein
